# Characterization of the Percival detector with soft X-rays

**DOI:** 10.1107/S1600577520013958

**Published:** 2021-01-01

**Authors:** Alessandro Marras, Jonathan Correa, Sabine Lange, Vahagn Vardanyan, Tim Gerhardt, Manuela Kuhn, Frantisek Krivan, Igor Shevyakov, Manfred Zimmer, Moritz Hoesch, Kai Bagschik, Frank Scholz, Nicola Guerrini, Ben Marsh, Iain Sedgwick, Giuseppe Cautero, Dario Giuressi, Gregori Iztok, Ralf H. Menk, Martin Scarcia, Luigi Stebel, Tim Nicholls, William Nichols, Ulrik K. Pedersen, Polad Shikhaliev, Nicola Tartoni, HyoJung Hyun, SeongHan Kim, KyungSook Kim, SeungYu Rah, Arkadiusz Dawiec, Fabienne Orsini, Giovanni Pinaroli, Alan Greer, Steve Aplin, April D. Jewell, Todd J. Jones, Shouleh Nikzad, Michael E. Hoenk, Frank Okrent, Heinz Graafsma, Cornelia B. Wunderer

**Affiliations:** a Deutsches Elektronen-Synchrotron, Notkestraße 85, Hamburg, Germany; b Center for Free Electron Laser Science (CFEL), Hamburg, Germany; c Science and Technology Faculties (STFC), Rutherford Appleton Laboratory (RAL), Didcot, United Kingdom; d Elettra Sincrotrone Trieste, Trieste, Italy; e INFN Trieste, Trieste, Italy; f University of Saskatchewan, Saskatoon, Saskatchewan, Canada S7N 5A2; g Diamond Light Source (DLS), Didcot, United Kingdom; h Pohang Accelerator Laboratory (PAL), Pohang, Gyeongbuk 37673, Republic of Korea; i SOLEIL Synchrotron, Saint Aubin, France; j Instrumentation Division, Brookhaven National Laboratory (BNL), Upton, NY 11973, USA; k Observatory Sciences Ltd, Cambridge, United Kingdom; l European XFEL GmbH, Schenefeld, Germany; m Jet Propulsion Laboratory, California Institute of Technology, Pasadena, CA 91109, USA; n Cycle GmbH, Hamburg, Germany; o Mid Sweden University, Sundsvall, Sweden

**Keywords:** Percival, detector, soft X-rays, CMOS Imager, photon science

## Abstract

In this paper the back-side-illuminated Percival 2-Megapixel (P2M) detector is presented, along with its characterization by means of optical and X-ray photons. For the first time, the response of the system to soft X-rays (250 eV to 1 keV) is presented. The main performance parameters of the first detector are measured, assessing the capabilities in terms of noise, dynamic range and single-photon discrimination capability. Present limitations and coming improvements are discussed.

## Abstract 2

Characterization of the Percival 2Megapixel detector with soft X-rays is presented.

## 1. Introduction   

The Percival collaboration was formed with the aim to provide the scientific community with a large pixelated detector, able to distinguish single photons in the soft X-ray regime, capable of large dynamic range, with a pixel pitch smaller than what is usual in hybrid assemblies and frame-rate high enough to allow single-shot experiments in most free-electron lasers (FELs). Combining these challenges results in a unique detection system, allowing to take full advantage of the luminosity improvements that FELs and diffraction-limited synchrotron rings (SRs) can provide (Price *et al.*, 2007 ▸; Prat *et al.*, 2007 ▸). The detector is particularly suited for photon science experiments in the water window (between the carbon and oxygen edges, 282–533 eV), and its main energy range also covers the transition-metal *L*-edges used in fast-demagnetization studies (Vodungbo *et al.*, 2016 ▸).

The collaborating institutions Deutsches Elektronen-Synchrotron (DESY), Science & Technology Faculties (STFC) Rutherford Appleton Laboratory (RAL), ELETTRA Sincrotrone Trieste, Diamond Light Source (DLS), Pohang Accelerator Laboratory (PAL) and Synchrotron SOLEIL together have developed an instrument matching those goals: the Percival detector.

Characterizations of small-size prototypes of the Percival detector have been presented in the past (Khromova *et al.*, 2016 ▸). Similarly, test results were reported on the front-side-illuminated (FSI) version of the full-scale detector (Marras *et al.*, 2019*a* ▸; Sedgwick *et al.*, 2019*a* ▸), with visible photons or tender X-rays used to overcome the thick front-oxide of the FSI version. In this paper, for the first time, we report on the performance of the back-side-illuminated (BSI; optimized for low-energy photons) version of the full-scale detector, as a response to soft X-rays in the detector primary energy range (250 eV to 1 keV).

This paper is subdivided into six sections. After this introduction, an overview of the system and its components is provided in Section 2. The measurement of performance parameters, estimated with the use of visible and soft X-rays photons, can be found in Sections 3 and 4. A summary of the detector main performance parameters is presented to Section 5, where we also discuss selected possible applications in scientific experiments, present limitations, and strategies to overcome them. Conclusions are summarized in Section 6.

## 2. The Percival system   

**Figure 1:**
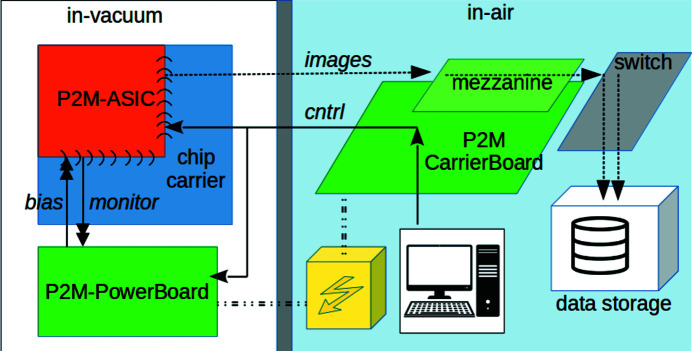
The P2M system: abstract description of the detector structure.

The Percival system is composed of a photon-sensitive element, hosted in a detector-head assembly, a control section that interfaces with the user, a data acquisition (DAQ) system and a common power supply. A global scheme of the system is visible in Fig. 1 ▸, and a description can be found in Wunderer *et al.* (2019 ▸). The individual components of the system are described in detail in the subsections of this chapter.

As a general-purpose photon imager, the photon-sensitive element (Sections 2.1–2.3) is able to operate in air, but for our target energy range vacuum operation is advisable: either integrated in a beamline setup, or in a a dedicated vacuum vessel (see Fig. S1 of the supporting information). The in-vacuum detector head is biased by a dedicated board (Section 2.4) and receives instructions from an in-air control system (Section 2.5). A data-concentrator board converts the data flow to a data protocol suitable for network packet exchange (Section 2.6), and passes it to a DAQ system (Section 2.7) able to record the images on disk in hdf5 format, in real time. The control interface is described in Section 2.8.

### 2.1. The ASIC (imaging sensor)   

The ASIC is a monolithic active pixel sensor, manufactured in a commercial 180 nm technology. It includes a 2-million-pixel array and peripheral circuits to digitize and streamout the data in LVDS format. A top-level overview of the sensor is shown in Fig. S2 of the supporting information.

**Figure 2:**
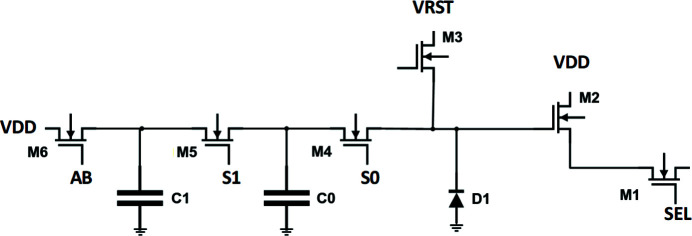
ASIC: pixel schematic.

The imaging area is a 1484 × 1408 array of LOFIC-type pixels [lateral overflow integration capacitor (Sugawa *et al.*, 2005 ▸; Wang *et al.*, 2001 ▸)], having a pixel pitch of 27 µm. The pixel architecture was chosen as it allows high-dynamic-range imaging for pulsed signals (such as the ones coming from a FEL). As shown in Fig. 2 ▸, the pixel includes a photodiode and two overflow capacitors. The pixel pitch was chosen as a reasonable trade-off between pixel density and full-well depth. An array of 1484 × 32 reference pixels is included at the edge of the imaging array.

The architecture allows for a static definition of the sensor gain and dynamic range, by programming the M4 and M5 transistor to add the desired amount of capacitance to the photodiode node. Thus, if the expected illumination level is known *a priori*, the detector could be configured for the situation corresponding to the best trade-off between noise and full-well. Such operation mode will be referred to in the rest of the paper as fixed-gain operation.

The architecture also allows for a dynamic extension of the dynamic range, by adaptively changing the capacitance of the charge-collecting node (and thus the system gain) to suit the collected charge, independently pixel to pixel and frame to frame. Such operation mode will be referred in the rest of the paper as adaptive gain operation. In this operation mode, the diode, as well as the C0 and C1 capacitors, are reset to VRST using the transistor M3. The M4 and M5 transistors are biased near their threshold voltage. When illuminated, photo-generated carriers accumulate on the diode, reducing its voltage; once the diode voltage has fallen low enough to turn on the M4 transistor, further carriers reduce the voltage on the C0 capacitor. Eventually, the M5 transistor is also turned on, and further carriers reduce the voltage on the C1 capacitor. Once illumination is complete, each diode and capacitor is read out in turn. This allows small signals (which accumulate only on the diode) to be read out with a high conversion gain (and hence low read noise), whilst large signals can still be accumulated on larger capacitors, increasing the dynamic range. The selection of the relative sizes of these capacitors is critical for ensuring that the noise level of the sensor remains below the shot noise of the incident light as the gain level switches (Akahane *et al.*, 2009 ▸).

Further improvement in noise performance for the high conversion gain mode is achieved by making use of correlated double sampling (CDS), which removes kTC noise introduced when resetting the pixel, by reading the pixel value before and after the signal has been collected. Further reduction of the noise can be obtained by the gain amplification provided by a programmable gain amplifier (PGA) circuit in the sampling stage.

The readout of the pixel array happens in CMOS-imager fashion. To permit a high frame-rate, seven rows of the sensor are read in parallel, and converted at the bottom of the column by seven ADCs per column, operating simultaneously. An eighth ADC is available as spare.

When operated in adaptive-gain mode, the pixel output is evaluated by an internal decision block, in order to limit the task of ADC digitization to relevant information only. This internal decision block sequentially probes the signal level on the diode D1, and the capacitor, C0 and C1: only the first signal which is not saturated is passed on to the ADC for digitization. The decision block also outputs the corresponding gain setting as two-bit binary value.

The ADC circuit is dual-slope type: it performs a 5-bit coarse conversion followed by an 8-bit fine conversion, leading to a 13-bit overall result, which will be later evaluated as a 12-bit number (plus gain level). A characterization of the ADC circuit in terms of differential non-linearity (DNL) and integral non-linearity (INL) can be found in Sedgewick *et al.* (2019*b* ▸). Data are transmitted off-chip using 45 LVDS data lines. Each LVDS line services 32 columns, each of which contains 7 gain-handling/ADC blocks, generating 15-bits per readout pixel. The resulting 3360-bit data packets are serialized through an on-chip serializer per LVDS line. The serializer is driven by a clock generated from a slower input clock by an on-chip phase locked loop (PLL).

The readout is configurable, and the imaging area can be polled in different ways, optimized for FEL or synchrotron radiation operation. For the purpose of the measurements presented in this paper, the imaging area was polled in a modified rolling-shutter fashion, that differs from classical rolling-shutter as it also contains a time window where all the pixels are integrating charge at the same time.

The estimated power consumption of the ASIC is of about 10 W. Details on the chip design can be found in Marsh *et al.* (2014 ▸).

### 2.2. Post-process for soft-X-ray applications   

Commonly used materials for silicon-surface passivation (SiO2 and Si3N4) have low transparency for photons in our target energy range and would reduce substantially the detector efficiency. Entrance window minimization is therefore paramount for our goals. This rules out a FSI architecture – where the back-end-of-line (BEOL) dielectric would be between incoming photons and the sensitive silicon – and moreover it demands minimization of inert material on the entrance surface of a BSI device. Surface quality is also important, as low-energy photons generate carriers near the entrance surface: trap-rich regions near the surface would get those carriers trapped/recombined near the generation point. For the same region, zero-field regions (such as thick wells of heavily doped material) should be avoided. Further constraints come from the limited voltage range of CMOS sub-micrometre nodes, which would not allow good depletion of thick volumes: the sensitive silicon is therefore to be thinned. The activation of the back-junction must also avoid high temperatures (commonly used in oven-annealing), as they would compromise the BEOL stack of the monolithic device.

To handle these constrains, we chose the following post-processing procedure.

The ASIC manufacturing occurs on wafers with a thick epitaxial layer of high resistivity. The chip is coupled BEOL-first to a handling wafer (using direct bonding): the silicon back-side is thinned (by a combination of grinding, etching and chemical-mechanical polishing to minimize reticle defects) to a thickness of about 10 µm, exposing the high-resistive epi-layer.

For the critical process of forming the back p-junction, we adopted the delta-doping process (Hoenk *et al.*, 1992 ▸, 2009 ▸, 2014 ▸; Nikzad *et al.*, 2012 ▸, 2017 ▸) developed and provided by Jet Propulsion Lab, consisting in a low-temperature molecular beam epitaxy, which grows a layer of silicon (including a shallow doped junction) on the thinned back-surface of the device. The layer is grown with the dopant in substitutional position, so it is not necessary to go through activation steps that would risk damaging the BEOL (high-temperature oven annealing) or introducing reticle defects near the surface (laser annealing). The silicon and silicon oxide covering the pads is then removed, so that pads can be contacted through wirebonding from the back-side.

The process itself is well established; for Percival it has been tested in the past on reduced-size prototypes, and the detectors treated in this way have shown good results for low-energy photons (Marras *et al.*, 2019*b* ▸); here we are presenting for the first time the performance of a full-scaled Percival device. Alternate processes are being explored as well, aimed at providing solutions that could be used for higher, less challenging, energies, in exchange for a less complicated process. Such alternate solutions will be covered in future publications.

### 2.3. The P2M cold head   

The ASIC is integrated into the ‘cold head’, comprising signal redistribution as well as mechanic thermal interfaces. A custom low-temperature cofired ceramic board (LTCC) (https://www.koaglobal.com/product/category/ltcc) brings the electrical signals (connected to the chip pads by wirebonding) to a set of standard pluggable connectors. The LTCC is held in a fixed position with respect to the chip by a set of milled brackets in polyether-ether-ketone (PEEK). Exposed devices are covered to minimize e.s.d. accidents, and the wirebond area is masked by a frontal 3D-printed metal cover (Fig. S3 of the supporting information). An aluminium frame on the back provides the attachment point for the detector in its intended position and fixing points for the cables and the P2M-PowerBoard described in the next section.

While the ASIC is capable of operation at room temperature, for optimal performance it is cooled down to −20°C. The ASIC is glued to a molybdenum support with thermally conductive glue, and screwed to a copper block. This section of the assembly is connected to a cooler by means of a copper path. The LTCC, PEEK brackets and aluminium frame are kept at higher temperature: they have only limited contact to the cold parts of the head, to reduce the thermal losses.

### 2.4. The P2M-PowerBoard   

The P2M-PowerBoard is a 103 mm × 239 mm ten-layer PCB, dedicated to power supply, biasing and monitoring for the P2M sensor. The power board provides 24 voltages and 21 bias currents: all are independently controllable (for fine-tuning to the optimal operating point of the detector) and can be live-monitored.

Live monitoring is also available for diagnostic purposes on external power lines, and remote temperature sensors allow probing the temperature behaviour of the detector head and its surroundings during operation, through 12 temperature channels. The organization of voltage and current sources on the board follows a specific symmetry such that the board can match either the FSI or the BSI configurations.

The power board has proven to work reliably in vacuum.

In order to maximize thermal contact to a cooling plate and to ease debugging, all the active components are placed on a single board side: this called for a dense board layout and tight mechanical constraints. At the cost of cutting some diagnostics and/or redundant off-sensor biasing, there is a significant margin to simplify and reduce the size of the power board in the future.

### 2.5. The P2M-CarrierBoard   

The P2M-CarrierBoard is responsible for the coordination of all the elements in the custom hardware system, from the sensor to the DAQ.

The P2M-CarrierBoard embeds a Virtex-6 FPGA, which is controllable via an Ethernet connection, as well as a suitable buffering for the input signals, coming from the P2M detector head through high-density twinax cables. Special attention has been taken to achieve net length equalization of such signals (which are mostly differential). On the DAQ side, the P2M-CarrierBoard hosts, supplies power to, and controls the data concentrator (mezzanine) board described in the next section.

In addition, the board has dedicated slots for two optional piggy-back boards dedicated to retrieving the facility-specific information (such as bunch-number ID), to synchronize the acquisitions and improve the integration of the system into the beamline environment.

The firmware of the Virtex-6 FPGA was developed in Verilog HDL. High flexibility has been required for the implementation of the readout logic, allowing on-the-fly adjustments for clock frequencies, signal timing, and acquisition modes. All settings are mapped to a distributed memory inside the FPGA, which is controlled via Ethernet with a full duplex UART protocol.

Additional firmware features include: automatic monitoring of the values provided by the P2M-PowerBoard (combined with the capability of issuing internal alerts and of performing basic safety actions), support of region-of-interest (ROI) readout, different triggering options, a basic custom protocol to interface with the piggy-back boards and an SPI communication for the slow control of the data concentrator board.

### 2.6. The data concentrator board (mezzanine)   

The imaging array is streamed out of the P2M-Cold Head via 45 LVDS pairs, along with auxiliary clocks and strobes. The data concentrator board reduces these multiple parallel signals to a sequence of standard transmission packets in UDP format, with fixed payload size (4.928 kByte) and unique header. One complete image (a ‘Sample’ and a ‘Reset’ acquisition, corresponding to data acquired just before and after the photon integration) corresponds to 1696 such packets, a total of 8.3 MB. Onward transmission is performed via two to three optical 10 Gb s^−1^ fibre links.

The data concentrator board features an on-board FPGA, memory banks, and the driver architecture for up to four 10 Gb s^−1^ links. Used for multiple projects, it is described in detail by Zimmer & Sheviakov (2012 ▸).

### 2.7. Percival data acquisition   

The data from the mezzanine card is passed to a cluster of Linux servers, via a deep buffer switch.

The function of the deep buffer switch is to reroute the data comprising one frame to the same server, according to the address provided in the unique UDP header. As the addresses are changed from frame to frame in a round robin fashion, the computing load is evenly distributed among the different Linux servers of the cluster. The Linux servers grab the frames, format the data properly, if desired apply the calibration parameters, and send the data to storage. Two parallel processes on a single server have been used for the measurements reported in this paper; we envisage to use four servers to acquire and process data of the detector at its maximum speed.

**Figure 3:**
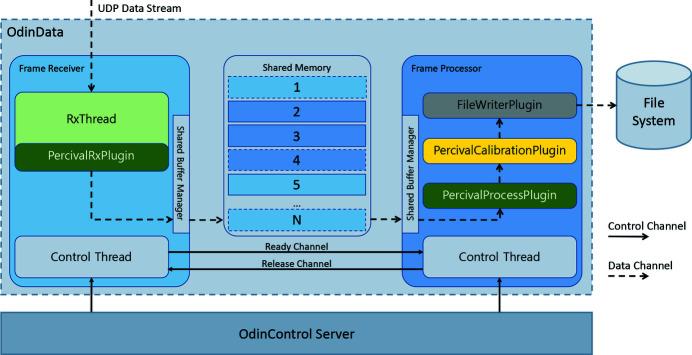
Odin-Data functional blocks and the data flow.

The data acquisition software which runs on each of the Linux servers is the OdinData framework (Yendell *et al.*, 2018 ▸). OdinData is a generic framework designed to be highly configurable into different patterns and states. Customization for Percival is done through plugins/adapters, catering to its specific requirements. Fig. 3 ▸ shows the functional blocks and the data flow specifically for Percival.

The frames from the detector are delivered to the frame receiver process, which creates a buffer of inter-process memory, and, when a UDP packet arrives, the process looks at the placement header, calculates the offset into the buffer accordingly, and copies the image data to this location. By this method a sequence of buffers are filled (one per image), by a custom Percival plugin to the Odin framework; when either a time-out is reached, or the image is full, the buffer is passed from the frame receiver to the frame processor process.

The frame processor has several plugins to treat the data received in the shared-memory buffer, including a ‘Process’ plugin (which accepts the data and releases the shared memory for reuse), a ‘Calibration’ plugin (under development, which transforms the raw output of the sensor in a meaningful physical parameter), a live-viewer utility, and a file-writer (that saves the frames in an hdf5 file).

Some details on the data acquisition backend is provided by Pedersen *et al.* (2014 ▸).

### 2.8. The P2M-Control interface   

The detector is controlled through a 1 Gbit s^−1^ Ethernet connection on the P2M-CarrierBoard. A process running on one of the servers sends the commands to the camera through the TCP/IP protocol.

The control software (OdinControl) provides a webserver which accepts standard http requests at specific urls, in the same way a web-browser fetches html pages from the internet and displays them to the user. The webserver runs on a host in the network: requests are routed by the webserver into a Percival adapter where Percival-specific code is available to interpret and respond to them.

There are several ways to send http requests to the webserver: either as a Python module (offering the user Python commands that could be combined in scripts), or as an html/javascript webpage suite (which sends http requests using javascript ajax and presents an html user-interface to the user).

The control of the detector head (through the carrier board TCP/IP connection) relies on a simple protocol embedded in the control firmware: the data specify an address and a value and the firmware will read/write the register at that address and thus parameters of the detector will be altered. A lookup table provides the list of specific register-name to address, thus interpreting the command.

Finally, the control system includes a way to communicate with frame processors and frame receivers, so the webserver can be used to query the status of these, and to send them configuration commands.

## 3. Characterization of the system operating with statically programmed (fixed) gain   

In order to interpret the detector outputs in terms of meaningful physical parameters, extraction of preliminary calibration parameters is necessary. Calibration does not require access to an X-ray source: it can be performed either with internal calibration circuits or with visible light. The calibrations require a few hours of measuring time each, but they are highly automatized, and, once done, the parameters can be used for several months, without appreciable reduction of performance.

The basic steps consist of:

(1) ADC-calibration, in which the transfer function is extracted for each of the on-chip ADC circuits. An internal calibration circuit (able to provide a known voltage at the input of the ADC) allows to measure their response in parallel.

(2) Fixed-gain calibration, that determines how to convert ADC outputs to meaningful physical parameters (electrons collected by the photodiode). The photon transfer curve (PTC) method, described in detail by Janesick (2007 ▸), is used for this purpose. The procedure requires exposing the array to a constant photon flux (and is eventually repeated at different intensities). A photon source in the visible regime (such as a LED of well defined wavelength) can be used for this purpose.

Some key-performance parameters (noise, full well, *etc*.) can also be extracted from the same datasets used to extract the calibration parameters: they will be described below, and then summarized in Table 1 in Section 5.

### 3.1. Full well and dynamic range   

As explained in Section 2.1 the system is configurable. When working in fixed-gain mode, an optimal trade-off between noise and full-well can be chosen, by statically programming the behaviour of the pixels transistors and the PGA circuit for the whole array. One out of four possible e/ADU ratios can be selected in advance, which in the following will be referred to as:

(i) very high gain (in which only the photodiode is used to integrate charge, maximizing the system response to charge collection; the system gain is further improved by amplification provided by the PGA circuit);

(ii) high gain (as above, only the photodiode is used to integrate charge, but the PGA circuit is now disabled);

(iii) medium gain (the smaller overflow capacitor is connected in parallel to the photodiode, increasing the full well at the expense of the system gain);

(iv) low gain (both overflow capacitors are connected to the photodiode, maximizing the full well).

The e/ADU ratio measured for each modes is reported in Table 1: the values vary logarithmically from a minimum of 2.1 e/ADU in very-high-gain mode to a maximum of 944 e/ADU in low-gain mode.

**Figure 4:**
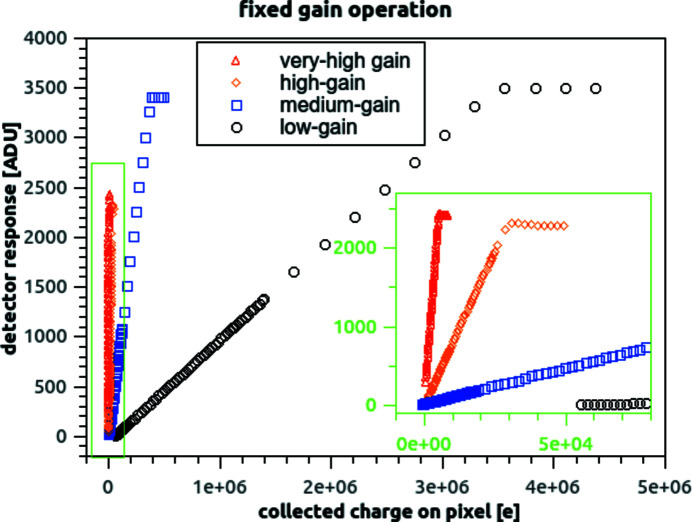
Response of the detector to constant flux and increasing integration time, in different operating modes: exemplary response of a single pixel, operating mode suppressing cross-talk. The lateral overflow circuit is disabled for static gain operation in one of the gain modes. A zoomed-in detail of the response curves is reported in the green inset, so that the response in high- and very-high- and medium-gain mode can be distinguished. The region shown corresponds to the region marked with a green rectangle in the overall graph.

Higher gain modes significantly reduce the system noise (as it will be shown in the next subsection), at the price of an earlier saturation of the system output, and thus of a linear full-well reduction. An example of the static (fixed gain) dynamic range capabilities is shown in Fig. 4 ▸. Full-well values were extracted corresponding to the point where the ramps start saturating (deviation by more than 2% with respect to the expected linear behaviour). Full-well values increase as the detector gain decreases: the values vary logarithmically from a minimum of 5.75 ke (very high gain mode), to a maximum of 3.56 Me (low-gain mode), and are reported in Table 1.

### 3.2. Noise   

The noise of the detector can be calculated from dark datasets. Given a set of images (typically 500–1000), acquired in dark condition, with the same integration time and at a stable temperature, the r.m.s. noise at the detector output is calculated by taking, for each pixel, the standard deviation of the detector output. This is converted to an equivalent noise charge (e.n.c.) at the input by applying the e/ADU ratios mentioned above. To measure the results discussed in this section, cross-talk effects have been suppressed by operating operating in interleaved mode as described in Section 5.1.

**Figure 5:**
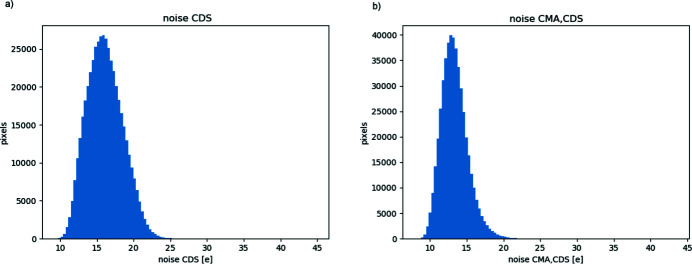
Distribution of input-referred e.n.c. among pixels (standard deviation of pixel output in dark condition, referred to equivalent input charge), very-high-gain mode, operating mode suppressing cross-talk: (*a*) correlated double sampling; (*b*) correlated double sampling and common mode averaging.

The noise of the detector depends substantially on the system gain. In low-flux conditions, the correlated ‘Sample’ and ‘Reset’ images (provided at the detector output) are to be digitally subtracted: this operation of correlated double sampling (CDS) reduces significantly the non-uniformities in dark images and, more importantly, suppresses reset noise and slow-signal variations (due, for example, to slow bias shift). The average noise after CDS is measured as 16.1 e [Fig. 5 ▸(*a*)].

A common mode component to the variation of the output of pixels in the same row can be suppressed, *e.g.* by subtracting a reference value (that can be estimated from a suitable group of reference pixels belonging to the same row). If such a common mode averaging (CMA) procedure is applied, our measurements show an improvement on the average noise level, that is reduced below 15 e [Fig. 5 ▸(*b*)].

Given the large dimension of the pixel array, some pixel-to-pixel variation of noise over the full sensor area is to be expected. From the histograms in Fig. 5 ▸, it is easy to see that most of pixels tend to have a similar value, with only small ‘tails’ of noisier and less-noisy pixels. Pixels nearer the edges of the detector were consciously excluded in this analysis, as the non-uniformity effect discussed in Section 5.4 makes them intrinsically different from the bulk of the pixel array. The presented analysis covers pixels in the central region of the array, from column 350 to column 1100 of the detector (*i.e.* roughly half of the imaging area).

**Figure 6:**
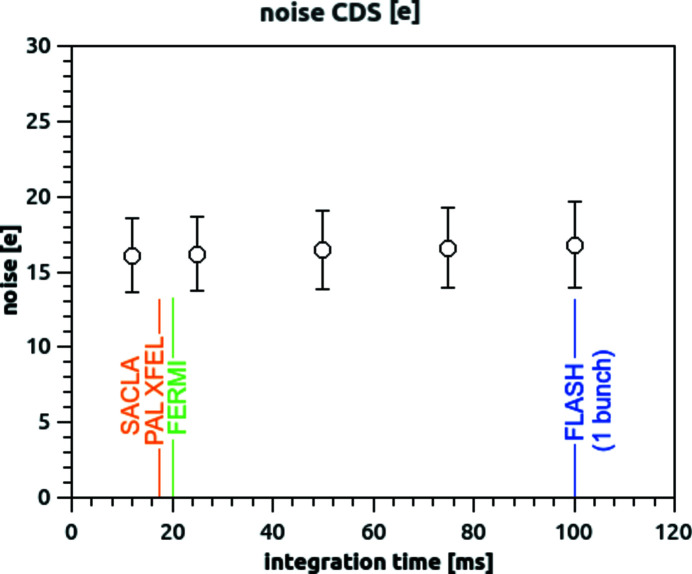
Measurement input-referred e.n.c. (std of pixel output in dark condition, referred to equivalent input charge), as a function of the integration time, in very high gain mode, operating mode suppressing cross-talk. Correlated double sampling is applied. The average noise value among the pixels reported in the graph as a circle, while the error bars represent the width (standard deviation) of the distribution.

All the data reported above have been measured at an 83.3 frame s^−1^ imaging rate (12 ms integration time). By repeating the same procedure described above, at different integration times (12–25–50–75–100 ms, corresponding to frame-rates of 83.3–40–20–10 frame s^−1^) we obtain the data reported in Fig. 6 ▸. It could be observed that the noise level remains very similar in the range, which covers the repetition rate of several soft FEL sources. All the measurements have been performed at a sensor temperature of −20°C (measured on the Cu block): we expect dark current to play a larger role in noise (and thus worsening performance for lower frame rate), when using the system at warmer temperatures.

If the gain is reduced, the noise increases because of the lower amplification factor. In addition, for medium- and low-gain modes, it is not always possible to operate a CDS elaboration of the data. The noise in the various gain stages are reported in Table 1.

### 3.3. Response to soft X-ray photons   

In order to test the detector response to soft X-rays in our target energy range (250 eV to 1 keV), we brought the system to the Variable Polarization XUV beamline P04, at the PETRA III synchrotron storage ring. Some details about the Variable Polarization XUV beamline P04 can be found in Viefhaus *et al.* (2013 ▸).

A retractable manipulator, 2.155 m upstream of the detector, has been used for the insertion of a pinhole. A sketch of the setup is shown in Fig. S4 of the supporting information.

When imaging soft X-ray photons coming from a beamline not completely free of higher harmonics, it is not straightforward to verify that an integrating detector response is not in part due to higher harmonics of the undulator radiation. Even small fractions of higher-harmonics photons in the beam could dominate the recorded signal and produce a false response from a detector, if the detector for some reason is blind to the main harmonic: we had observed this effect in the past (Wunderer *et al.*, 2014 ▸) when dimensioning the photodiode on prototype samples.

**Figure 7:**
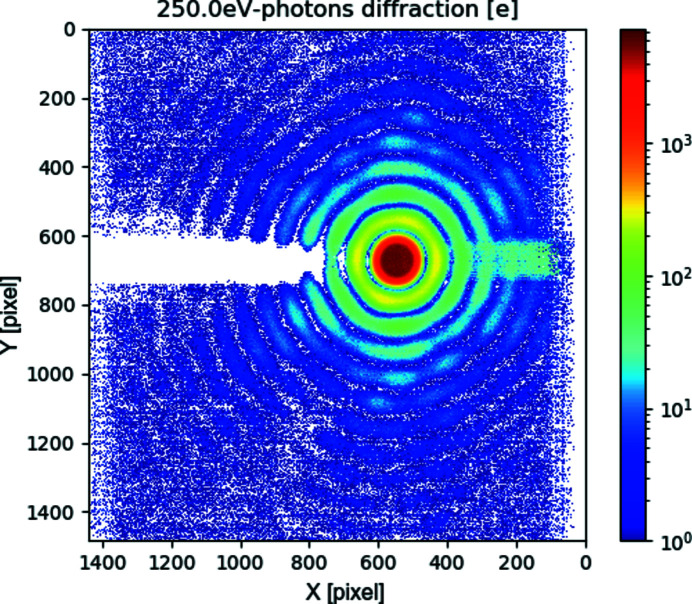
Soft X-ray (250 eV) diffraction through a circular pinhole: fixed (very-high) gain operation. Note that the top of the main peak is limited by the maximum full-well in this mode (a few thousand electrons). The horizontal stripe artefact related to the region of maximum illumination is discussed in Section 5.3

To ensure higher harmonics play no noticeable role, we have recorded the diffraction pattern from the circular aperture (pinhole) interposed between the beam and the detector: the diffraction produces (Fig. 7 ▸) a series of rings (Airy rings) (Hecht, 1975 ▸) whose radial frequency is a function of the wavelength. By measuring the ring frequency – knowing the aperture diameter and distance – we can determine the photon wavelength producing the spatial pattern of collected charge.

**Figure 8:**
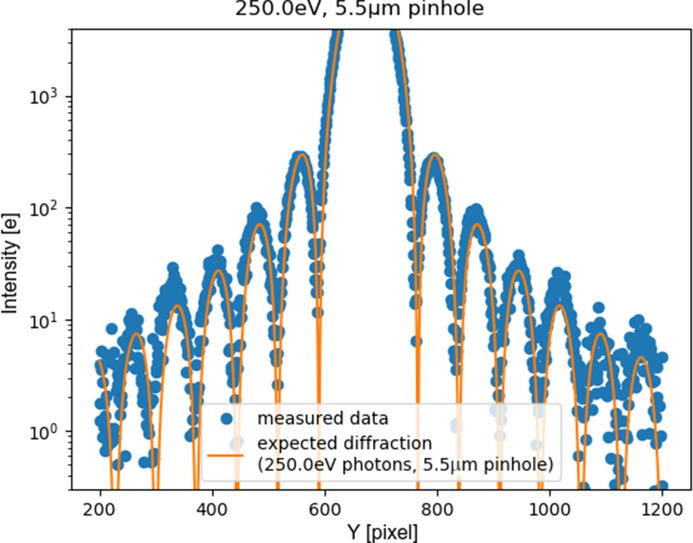
Cutplane of Airy ring pattern: fixed (very-high) gain operation. Comparison between detector output and theoretical prediction for soft X-rays (250 eV photons diffraction through a circular pinhole). The good matching of the measured and expected spatial frequency of the rings suggests that the detector output is dominated by photons of the nominal energy, rather than by higher harmonics photons. Note that the top of the main peak is limited by the maximum full-well in this mode (a few thousand electrons).

Fig. 8 ▸ compares the system response with the mathematical prediction of the pattern, for the nominal wavelength, distance and pinhole diameter. The good match of the prediction to the data confirms the detector output being dominated by main harmonic photons for lower energy photons (250 eV).

Our best-fit parameter for the pinhole diameter (5.5 µm) is 10% larger than the nominal diameter: this deviation was considered in line with the accuracy limitations on the nominal value estimation, and in any case does not impair the consideration about main harmonic photons being dominant. Similar good matching to the mathematical prediction were obtained for higher (399 eV, 710 eV, 1000 eV) energy photons.

This successful comparison rules out the presence of a thick inert material layer on, or a trap-rich region near, the sensor surface. The former would have prevented (main harmonics) low-energy photons from reaching the silicon, while the latter would have prevented carriers (generated by such photons near to the surface) from being collected. In both cases, the sensor output would have been dominated by higher harmonics components rather than the photons belonging to the main harmonic.

### 3.4. Signal-to-noise ratio and single-photon sensitivity   

**Figure 9:**
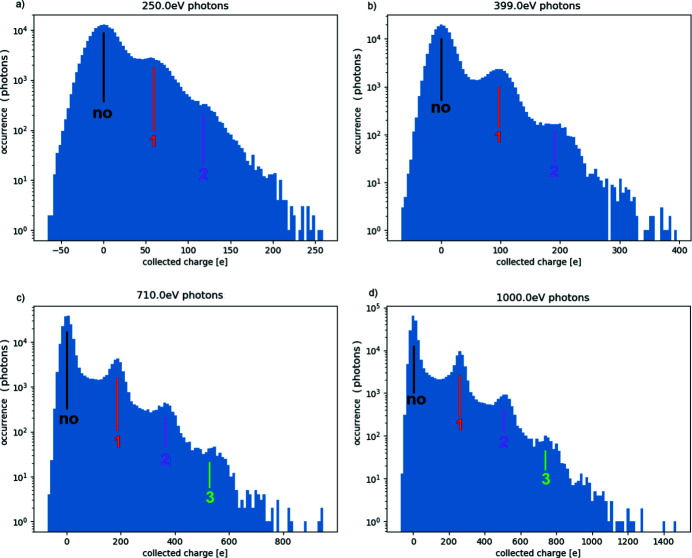
Spectrum measurements for several photon energies (very high gain mode, CMA, operating mode suppressing cross-talk): (*a*) 250 eV photons; (*b*) 399 eV photons; (*c*) 710 eV photons; (*d*) 1000 eV photons. Histograms are reported in log scale, to allow recognition of the multiple-photons peaks.

In order to evaluate the single-photon discrimination capability of the detector, a region about 10 × 10 pixels, illuminated at suitably low flux, has been selected. Histogramming the per-frame signal from these pixels into one common histogram results in a series of Gaussian peaks, regularly spaced along the horizontal axis. We interpret the leftmost of these peaks (centred around an average charge collection of 0 e) as the detector response to a dark situation (‘noise peak’), *i.e.* the situation in which no photon arrives on the pixel. We interpret the peaks to the right of the ‘noise peak’ as the detector response to, respectively, one photon, two photons, three photons, *etc*. Some examples of photon spectra in the 250 eV to 1 keV energy range are shown in Figs. 9 ▸(*a*)–9(*d*). To measure the results discussed in this section, cross-talk effects have been suppressed by operating in interleaved mode as described in Section 5.1.

**Figure 10:**
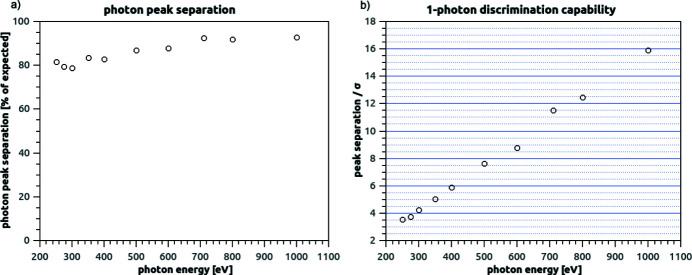
(*a*) Measurements of ratio of collected charge for different energies in the 250 eV to 1 keV range (very high gain mode, CMA, operating mode suppressing cross-talk). The RCC is defined as the ratio between ‘photon peak’ separation (*i.e.* charge collected by a pixel) and the expected charge generation (under ideal conditions). (*b*) Single-photon discrimination capability: SNR at different energies in the 250 eV to 1 keV range (very high gain mode, CMA, operating mode suppressing cross-talk).

For all the photon energies examined, the ‘photon peaks’ are centred on an average charge-collection value that is very similar (78–93%) to the total amount of charge that would be generated in silicon (under ideal conditions) by photons of that energy [Fig. 10 ▸(*a*)]. This ratio between ‘photon peak’ separation (*i.e.* charge collected by a pixel) and the expected charge generation (under ideal conditions) is referred as the ratio of collected charge (RCC) in the following. The measured RCC is slightly lower for lower energies, and approaches unity for higher energies. We interpret this effect as a consequence of lower energy photons, on average, generating charge nearer the entrance surface (thus further away from the collection junction): in their drift towards the collection junction, the generated electrons have more opportunities to either be shared between pixels, or to recombine.

It is to be noted that the data shows the raw response of each pixel, and no correction or clustering has been applied to compensate for possible charge sharing among adjacent pixels: we expect that some improvement might come from such compensation.

The standard deviation extracted from noise peaks is consistent with the noise values reported in Section 3.2. Thus the single-photon discrimination capability of the system can be calculated in terms of the signal-to-noise ratio (SNR), where the peak separation represents our signal and their standard deviation represents the noise [Fig. 10 ▸(*b*)]. As expected, the SNR is linearly increasing with the photon energy. The data shows that a SNR > 3 is achieved over the whole energy range, and that the system SNR is higher than 5 for 350 eV photons (and higher energies).

Only the electronic noise was considered as an error source for these estimations, because it is largely dominant over the Fano fluctuations (and the two components sum in quadrature). A more precise estimation of the energy resolution, also taking into account the Fano fluctuation in silicon, would increase the noise from the measured 16.1 e r.m.s. to 16.34 e r.m.s. (or 58.82 eV) for 250 eV photons.

For single-photon sensitivity, we adopt the definition proposed by Becker *et al.* (2012 ▸), as having a false positive rate below 10^−6^ (less than a false positive in a million pixel), while at the same time having a true positive rate >50%:
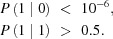
Using our noise measurements, we can model the noise as a Gaussian-distributed random variable, having an average of μ = 0 and a standard deviation σ equal to the measured e.n.c. An estimation of the expected fraction of false positives can be calculated as the integral under the curve from *T* to infinity, where *T* is the decision threshold used to evaluate the detector output. In other words,
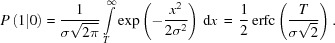
From the true positive constraint (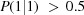), it can be derived that the decision threshold *T* must be lower than the median detector response to a single photon (as the measured one-photon peak also has a Gaussian distribution): we use the data measured to determine the photon peak average (as the constraint to *T*) in each case, inclusive of the RCC reduction shown in Fig. 10 ▸(*a*).

A suitable decision threshold *T* can be found to fulfil the false negative constraint (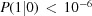) for photons of energy 350 eV and above. Therefore, we claim single-photon sensitivity starting from that energy level.

There may be some margin for improving this number, reducing further the noise level by means of a better suppression of the common-mode variation, as it is shown in Section 3.2.

### 3.5. Quantum efficiency estimation   

The ratio reported in Fig. 10 ▸(*a*) can give an estimation of the fraction of the charge generated in silicon, that is actually collected by the photodiode for a detected photon.

This datum alone is not enough to give an estimation of the detector quantum efficiency, as it does not take into account that some photons may not photogenerate in the silicon at all (for example, because trapped in an inert front window), and the data reported would be restricted only to the photons able to pass the front window. A similar scenario might be constructed by postulating that the charge generated by some photons might be 100% recombined in a trap-rich silicon layer, and the data reported would be restricted to the charge generated by the other photons. We do not, at the moment, have a measurement to disprove said scenarios, and give a precise value of charge collection efficiency (CCE) and quantum efficiency (QE) of the BSI-processed P2M detector.

We can, however, give a likely estimation of the efficiency, based on CCE measurements that had been done on reduced scale prototypes (Correa *et al.*, 2016 ▸) in combination with the information from the Section 3.4. Said prototypes had undergone the same post-processing described in Section 2.2: thus, if there is an inert entrance window preventing some photons to reach the silicon, it should be similar in the prototype as in the P2M detector. The design of the photodiode has been optimized with the aim to improving its ability to collect charge in the P2M detector respect to the prototype (by means of larger junctions): thus, if there is a fraction of photons whose charge is completely recombined before being collected, such fraction is expected be the same or lower in the P2M detector respect to the prototype.

We can calculate the expected QE as[Chem scheme1] 
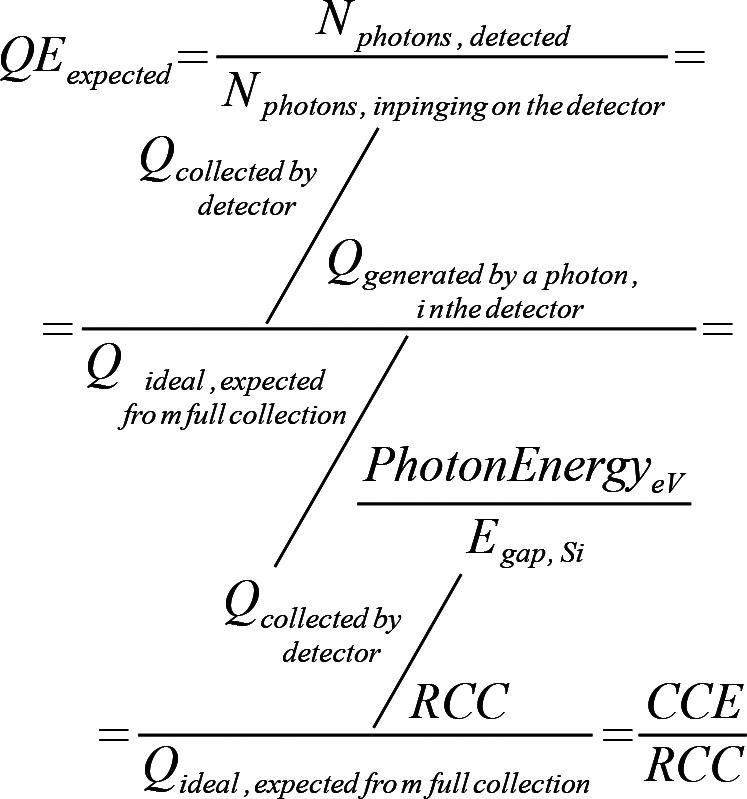


Here RCC < 1 is the ratio between the charge effectively collected (measured as peak separation in the photon spectrum) and theoretical charge generation in silicon caused by a photon of that energy.

**Figure 11:**
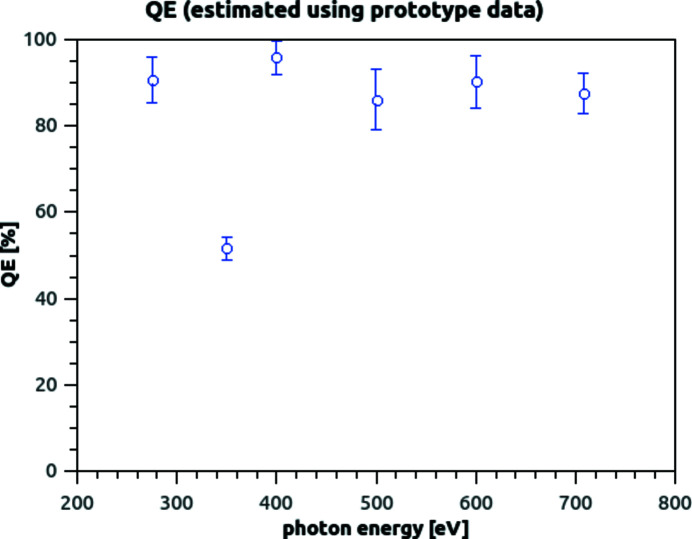
Expected QE values for the detector, using the CCE values measured on a reduced-scaled prototype and the RCC values measured on the P2M device. The average among ten measurement sets is reported in the graph as a circle, while the error bars represent the standard deviation. As explained by Correa *et al.* (2016 ▸), we think the efficiency reduction at 350 eV is due to accidental carbon contamination of the surface (absorption edge 282 eV).

We can use the CCE values that had been measured on reduced scale prototypes as likely figures of what we can expect of the P2M detector. If we combine the data of Correa *et al.* (2016 ▸) with the RCC values measured in Fig. 10 ▸(*a*), we can obtain an estimate of expected QE values for the P2M detector, reported in Fig. 11 ▸.

This is the best indication we can give on the basis of the data we have in hand at the moment: a measurement campaign will start soon to properly measure CCE and QE on the P2M detector.

## 4. Characterization of the system operating auto-adaptive gain (lateral overflow)   

The drawback of operating in static (fixed-gain) mode is that, if the incoming flux had been under-/over-estimated – or varies widely over the sensor area – there is the risk of the system response saturating, or having lower-than-anticipated signals drown in unnecessarily high noise. Enabling the lateral overflow circuit overcomes this problem by dynamically adjusting the gain to be used, pixel by pixel and frame by frame, to the incoming photon flux: so that, instead of reaching the saturation level, the charge will just be processed with a lower gain. In this operation mode, each pixel can choose to operate along three of the four possible e/ADU ratios described in the fixed-gain operation chapter (Section 3): the triplet of usable states is configurable. In the following, we explored the system behaviour for an auto-adaptive gain configuration using the triplet high/medium/low gain.

In order to interpret the detector output in this case, an additional calibration step is necessary (with respect to what is described in Section 3) to measure the detector transfer function over a wide range of charge-collection inputs. As for the PTC measurement, a photon source in the visible regime can be used for this purpose, and key-performance parameters can be extracted from the same datasets.

### 4.1. Full-well, switching points and noise   

**Figure 12:**
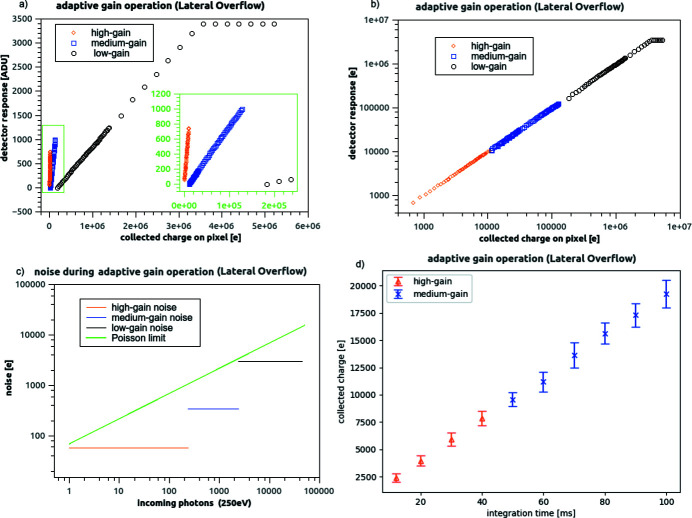
Detector operating in dynamical gain-adapting (lateral-overflow) mode: exemplary response of a single-pixel operating mode suppressing cross-talk. Each pixel decides independently which gain to apply to the collected charge, thus avoiding saturation. (*a*) Detector [ADU] response to an increasing collected charge. A zoomed-in detail of the response curves is reported in the green inset, so that the response in high- and medium-gain can be better appreciated. (*b*) Calibrated output (converted from ADU to electrons) for the same measurements. Several acquisition sets (using different attenuator filters) have been stitched together to evaluate the response along the full dynamic range: the uneven distribution of points along the overall curve is a consequence of the different fluxes (available illumination settings and filters chosen). (*c*) Comparison between circuit-induced noise levels and the Poisson limit. (*d*) Response to soft-energy (275 eV) photon exposure versus integration time (progressively increasing collected charge). The average among 1000 images is reported in the graph as an icon, while the error bars represent the standard deviation.

An example of adaptive gain operation is shown in Fig. 12 ▸(*a*). In a similar way to what is described for fixed gain (Fig. 4 ▸), the detector was exposed to a constant photon flux; data taken at a range of integration times allow to measure its response to an increasing level of collected charge. The process was repeated several times (using known attenuation filters to modulate the flux from the photon source by several orders of magnitude) to cover the full detector range.

The lateral-overflow circuit was enabled so that, for a given charge collection level, the system would change its status from high gain (optimized for low flux) to medium gain (optimized for medium flux), and then again to low gain (optimized for high flux). Thus the curve describing the relation between the collected charge and the system output consists of a succession of three ramps, having very different slopes. The data stream kept track of the applicable gain setting, encoding the lateral-overflow status in the first two bits of the detector output, independently for each image and for each pixel. Once calibrated (output converted from ADU to electrons), the detector output appears as in Fig. 12 ▸(*b*).

To verify that the lateral overflow circuit did not introduce a significant noise, e.n.c. noise was compared, between sets of dark images taken in fixed high-gain mode (disabling the lateral overflow circuit) and in auto-adaptive-gain mode (enabling it), while keeping all other system parameters constant: the measured average noise value did not change appreciably.

The switching points between high-, medium- and low-gain region have been chosen so that the system noise remains below the Poisson limit also for medium- and high-flux regimes [Fig. 12 ▸(*c*)].

### 4.2. Response to soft-X-ray photons (auto-adaptive-gain operation)   

We have tested the adaptive-gain acquisition mode by exposing the detector to a constant flux from the beamline, and taking several sets of images at different integration times. Thus charge collected by a pixel is expected to be a linear function of the integration time. We have chosen a combination of the incoming flux and integration time such that the collected charge was below the level triggering the adaptive-gain circuit for smaller integration times, and above that level for longer integration times. An example of pixel output (reconstructed using lateral overflow calibration parameters) is reported in Fig. 12 ▸(*d*). As expected, the detector response increases linearly with the integration time.

**Figure 13:**
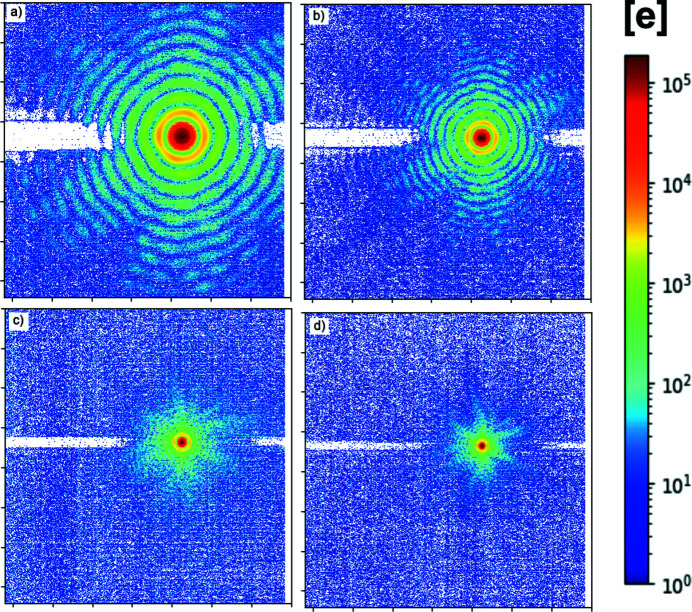
Soft X-ray diffractions through a circular pinhole, using photons of different energies: auto-adaptive-gain mode. (*a*) 250 eV photons; (*b*) 399 eV photons; (*c*) 710 eV photons; (*d*) 1000 eV photons. Airy ring patterns are visible in the detector output; as expected, the spatial frequency of the rings is proportional to the photon energy. The horizontal white stripe artefact (negative pedestal shift) related to the region of maximum illumination is discussed in Section 5.3.

**Figure 14:**
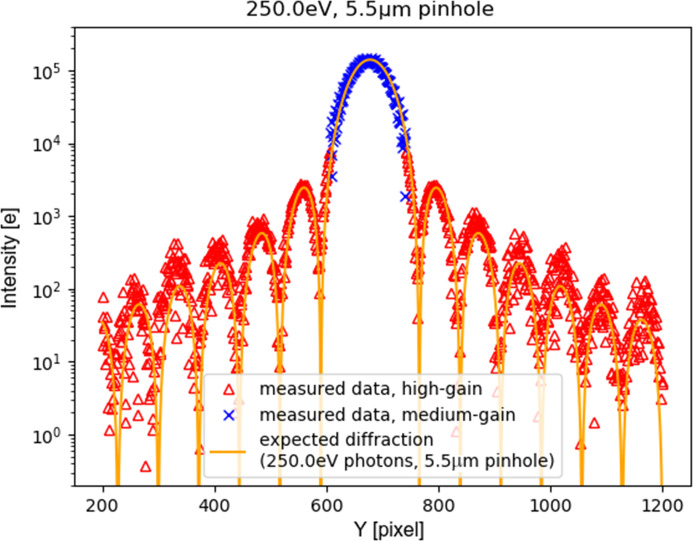
Cutplane of Airy ring pattern: auto-adaptive-gain operation. Comparison between detector output and theoretical prediction for soft X-rays (250 eV photons diffraction through a circular pinhole). Note that the top of the main peak is no longer limited to the full well of a fixed-gain operation mode, as it was for Fig. 8 ▸. Pixels amplified by high gain (red) and medium gain (blue data points) are shown with different colours to show the result of the auto-adaptive-gain modulation. Note that, because of parameter dispersion, each pixel has its own gain transition point, which results on some pixels reducing their gain earlier than others.

The effect can also be appreciated in an image that has both high-illuminated and low-illuminated areas. Some Airy patterns similar to the ones shown in Section 3.3 have been acquired in this adaptive-gain mode (Fig. 13 ▸). As expected (Fig. 14 ▸), the good match of the prediction to the data confirms the detector output being dominated by main harmonic photons. The dynamic range, however, is extended with respect to fixed-gain operation (Fig. 8 ▸), as the system now identifies the central peak of the innermost Airy disk as a medium-illuminated area, and thus lowers the gain of those pixels to avoid saturation. At the same time, the pixels recording peripheral Airy rings receive higher amplification, allowing precise measurement of low-signal peaks as well.

## 5. Considerations   

The main performance parameters for the systems are reported in Table 1 ▸.

Many soft X-ray experiments at high-luminosity beamlines can benefit by the detector capabilities. Ptychography and holography experiments, for example, can benefit by the frame rate (one to two orders of magnitude faster than many currently used CCDs). Also, in some fields (such as ptychography or CDI) the extended dynamic range of the detector can help to significantly improve the image resolution: methods commonly used today to reach higher resolution rely on artificially expanding the dynamic range, combining information from different acquisition sets obtained with different beamstop sizes (Rose *et al.*, 2018 ▸). Both issues could be tackled exploiting the Percival system frame rate and dynamic range. An exploratory experiment at P04 has tested Percival’s applicability to this field, with positive results, that will be covered in a future publication.

As mentioned, the system today has some fledgling limitations: we are outlining them here, together with the steps we are taking to solve those issues.

### 5.1. Elimination of cross-talk effects   

The ASIC has been designed to process the signals from the photodiode array in a pipeline fashion: while a pixel block *n* is being sampled, in parallel the data from the former block *n* − 1 is converted into the digital domain, and again in parallel the digitized data of block *n* − 2 is streamed out. Such a data-processing scheme exposes analogue signals to the risk of cross-talk from toggling digital ones, at the benefit of efficient – fast – operation

Unfortunately the current P2M chip is not free of such crosstalk. We have identified two sources of digital-over-analogue cross-talk aggressions in our ASIC, both acting on the analogue charge at the input of the ADCs.

We identified the first source as the toggling of signals controlling the sampling phase, and were able to completely remove this cross-talk, by temporally decoupling the aggressor signals, introducing a dead-time in the pipeline (at the cost of reducing the maximum frame rate down to 83.3 frames s^−1^ for the currently used streamout speed of 120 MHz).

A second cross-talk effect was identified as caused by a serialization signal: when not corrected, it causes a local deviation from linearity in the ADC that is not really visible on images covering a large dynamic range (like Fig. 7 ▸, Fig. 13 ▸), but might hinder measurements critically dependent on accurate detection of low-level signals, or near the gain-switching points. We were able to verify that the effect is suppressed if the aggressor is prevented from toggling. We have started implementing an alternate operation mode, that would decouple temporally also this second aggressor. For the moment we are suppressing this second cross-talk by preventing the digital signal from toggling. This approach has the unfortunate side effect that about half (4/7) of the digitized pixel values are not actually streamed out in this mode: it produces pictures where rows of data coming from the pixels are interleaved to apparently empty rows. We have used this mode of operation to evaluate parameters critically dependent on the noise level (in Sections 3 and 4).

One alternative approach we are exploring is to modify the linear ADC-calibration to a more complex algorithm, that would model the ADC response as a non-linear function. We are still exploring the effectiveness of such option: further details will be covered in a future paper.

Finally, a respin of the ASIC is foreseen, that will introduce proper counter-measures in the layout to avoid the cross-talk.

### 5.2. Improvements on frame rate   

As shown in Table 1 ▸, the system has been tested up to a frame rate of 83.3 frame s^−1^. This limitation on frame rate is due partially to the introduction of dead-times in the data-processing pipeline described in Section 5.1, and partially to the current use of a PLL clock (120 MHz) that is slower than what was originally envisioned for the detector (240 MHz).

The present frame rate would be enough to allow single-shot experiments in some free-electron lasers (FERMI, PAL-XFEL, SACLA, FLASH in single-bunch mode). We aim to improve the speed beyond 120 frame s^−1^, to extend the single-shot-experiment capability to more FELs (LCLS, SwissFEL). Synchrotron experiments would also benefit from a higher frame rate.

To speed up the frame rate, we have a variety of options that we are working on:

(i) the read-out of the detector in region-of-interest (ROI) mode. The pixel array is divided in 212 sub-sections that can be read out individually; the readout time is expected to scale down linearly with the number of sub-sections read;

(ii) the limitation of the ADC comparison to a lower number of possible digitized values;

(iii) a modification to the digital signal sequences, to avoid cross-talk during vulnerable phases while reducing the resulting dead-times in the pipeline;

(iv) the adoption of a faster PLL clock. A suitable firmware is being developed for the purpose;

(v) a chip redesign, that would prevent the cross-talk effects described (thus not having to introduce a dead-time in the pipeline, and doubling the frame rate).

We see no fundamental roadblocks to improving the frame rate substantially, and preliminary tests at 165 frame s^−1^ have been performed (without cross-talk suppression).

### 5.3. Signal-induced pedestal shift   

**Figure 15:**
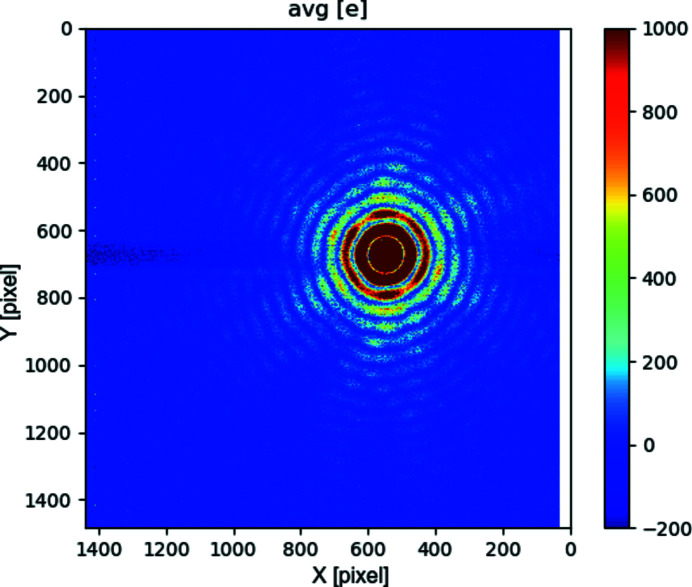
Response to localized high-flux: artificially reduced scale to make the pedestal shift (here affecting rows 620–700) noticeable, especially in the left part of the image.

When a region of the detector is exposed to a continuous high flux, we have observed a pedestal shift in pixels in the same rows as the highly illuminated pixels. The shift is different for different acquisition modes, and, for fixed (very high) gain mode, might be different on the two sides of the bright spot. In auto-adaptive-gain mode, we have measured this shift to be of the order of ∼200 e, for a peak signal of 0.3 Me pixel^−1^: it is not noticeable on a linearly scaled image, and only becomes apparent if the scale is artificially reduced (Fig. 15 ▸) or made logarithmic (Fig. 7 ▸, Fig. 13 ▸).

The pedestal shift happen in the direction perpendicular to the readout, involving the pixels that (in different columns) are read as the same time as pixels that are highly illuminated. We think it possible (but we cannot prove at this moment) that a supply or a bias might be experiencing a temporary voltage drop because of the current drawn by the circuits processing the signal of the highly illuminated pixels.

We are investigating the cause of this unexpected behaviour, and possible counter-measures.

### 5.4. Pedestal disuniformity   

**Figure 16:**
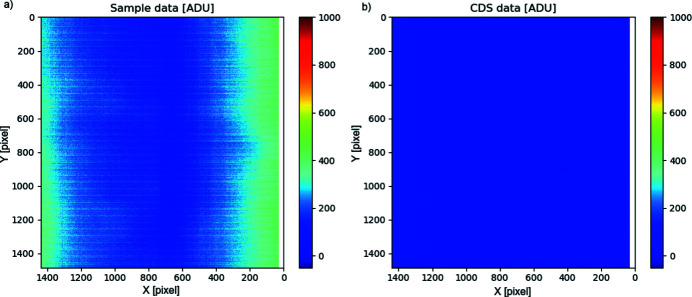
ADC response of the system to a flat-field illumination: fixed-high-gain operation mode. The baseline variation is the same for ‘Sample’ (*a*) and ‘Reset’ images, so the effect can be mostly suppressed by CDS (*b*). Further improvement in uniformity can be achieved by dark-image subtraction.

We have observed a non-uniformity in the device baseline (sensor response to an uniform dark condition): as a consequence, the outermost columns (both on the ‘right’ and on the ‘left’ of our imaging array) tend to respond with a ADC value that is higher than the value for pixels in the middle of the array [Fig. 16 ▸(*a*)], both for uniformly dark and uniformly illuminated images. This situation becomes more evident with increased gain. The baseline variation is the same for ‘Sample’ and ‘Reset’ images, and the measured pixel gain (e/ADU) remains uniform also near the edges, so a CDS operation (or even a dark-image-subtraction operation) brings the detector output back to an almost-uniform field-response [Fig. 16 ▸(*b*)]. The effect is not really visible in the images reported in Section 3, as it is suppressed by CDS or dark-subtraction.

However, this means that, given a uniform flat-field input that progressively increases, the output of the ADC on the ‘right’ and on the ‘left’ columns saturates earlier than the output of the ADCs in the middle of the image array, *i.e.* those regions have a lower dynamic range. The higher the gain used, the earlier the edges saturate: for very high gain, saturation at the edge can be reached already in dark conditions, preventing those pixels from operating with minimum noise.

While we recognize this limitation, it should be pointed out that the use of areas at a distance from the detector edges for critical measurements is a precaution that is far from being unusual.

We have at the moment too little statistics to make hypotheses on the cause of this effect, but we have observed it to be more apparent in one BSI device than in other FSI devices. The cause of this non-uniformity is under investigation.

## 6. Conclusions   

In this paper we have presented a comprehensive overview of the Percival detector, along with a characterization of its performance parameters in terms of noise, full-well, dynamic range extension (lateral overflow adaptive-gain), and single-photon sensitivity. We have focused in particular on the detector response to soft X-rays photons in its target energy range (250 eV to 1 keV). We have also discussed present limitations, and steps taken to solve them.

## Supplementary Material

Supporting Figures S1 to S4. DOI: 10.1107/S1600577520013958/ju5018sup1.pdf

## Figures and Tables

**Table 1:** Summary of the main performance parameters

Pixel array	2089472 pixels (+ references), 27 µm pitch
Frame rate	Tested: up to 83.3 frame s^−1^ (design goal: >120 frame^−1^ s^−1^)
e/ADU	Very high gain: 2.1 e/ADU
	High gain: 12.6 e/ADU
	Medium gain: 106.0 e/ADU
	Low gain: 944.2 e/ADU
Noise	Very high gain: 16.1 e ± 2.4 e (∼0.23 ph @ 250 eV) reduced <15 e by CMA
	High gain: 52–82 e ± 15 e (0.75–1.18 ph @ 250 eV)
	Medium gain: 343 e ± 73 e (∼4.95 ph @ 250 eV)
	Low gain: 3.0 ke ± 638 e (∼43 ph @ 250 eV)
One-photon sensitivity: *P*(1|0) < 10 × 10^−6^	350 eV photons and above (very high gain)
Full well (fixed-gain operation)	Very high gain mode: ∼5.75 ke ± 585 e (∼83 ph @ 250 eV)
	High-gain mode: 30.5 ke ± 2 ke (∼439 ph @ 250 eV)
	Medium-gain mode: 381 ke ± 17.6 ke (∼5.5 kph @ 250 eV)
	Low-gain mode: 3.56 Me ± 169 ke (∼51 kph @ 250 eV)
Adaptive-gain dynamic range (lateral-overflow)	High → medium gain: 16.4 ke ± 6.1 ke (∼236 ph @ 250 eV)
	Medium → low-gain: 165.5 ke ± 23 ke (∼2.4 kph @ 250 eV)
	Low gain (full well): 3.09 Me ± 201 ke (∼45 kph @ 250 eV)
