# Supplementary material for: Characterization of the Percival detector with soft X-rays
Source: J Synchrotron Radiat. 2021 Jan 1;28(Pt 1):131–45. doi: 10.1107/S1600577520013958 (PMC7842225; doi:10.1107/S1600577520013958)
Supplement: Supplementary file 1 [file s-28-00131-sup1.pdf]

JOURNAL OF SYNCHROTRON RADIATION

Volume 28 (2021)

Supporting information for article:

Characterization of the Percival detector with soft X-rays

Alessandro Marras, Jonathan Correa, Sabine Lange, Vahagn Vardanyan, Tim Gerhardt, Manuela Kuhn, Frantisek Krivan, Igor Shevyakov, Manfred Zimmer, Moritz Hoesch, Kai Bagschik, Frank Scholz, Nicola Guerrini, Ben Marsh, Iain Sedgwick, Giuseppe Cautero, Dario Giuressi, Gregori Iztok, Ralf H. Menk, Martin Scarcia, Luigi Stebel, Tim Nicholls, William Nichols, Ulrik K. Pedersen, Polad Shikhaliev, Nicola Tartoni, HyoJung Hyun, SeongHan Kim, KyungSook Kim, SeungYu Rah, Arkadiusz Dawiec, Fabienne Orsini, Giovanni Pinaroli, Alan Greer, Steve Aplin, April D. Jewell, Todd J. Jones, Shouleh Nikzad, Michael E. Hoenk, Frank Okrent, Heinz Graafsma and Cornelia B. Wunderer

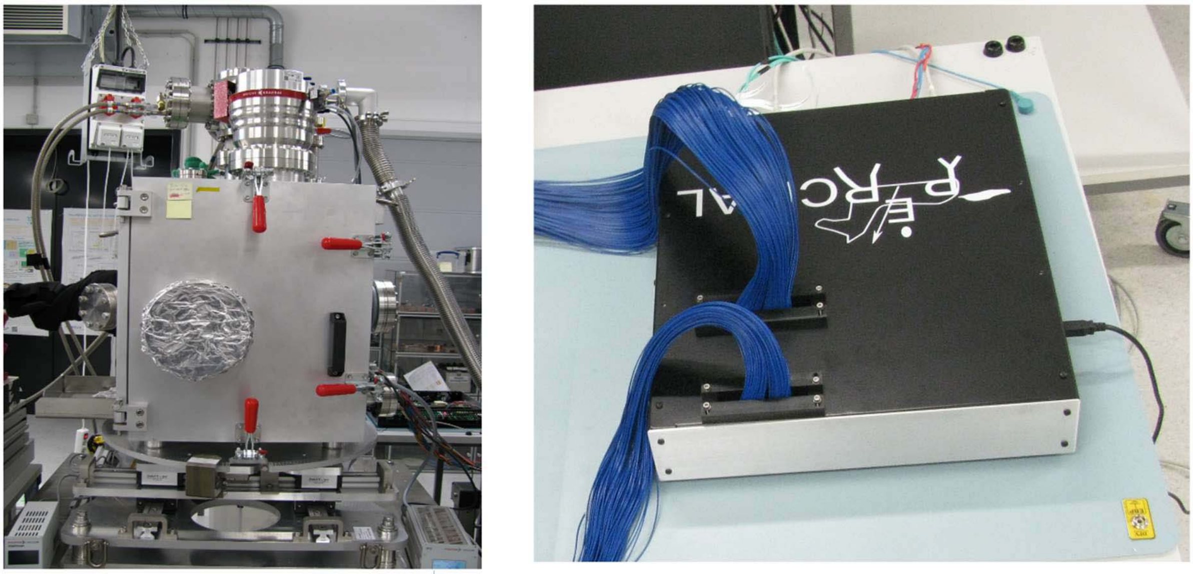

Figure S1 The P2M system in its actual components; vacuum vessel and (in-air) detector control box

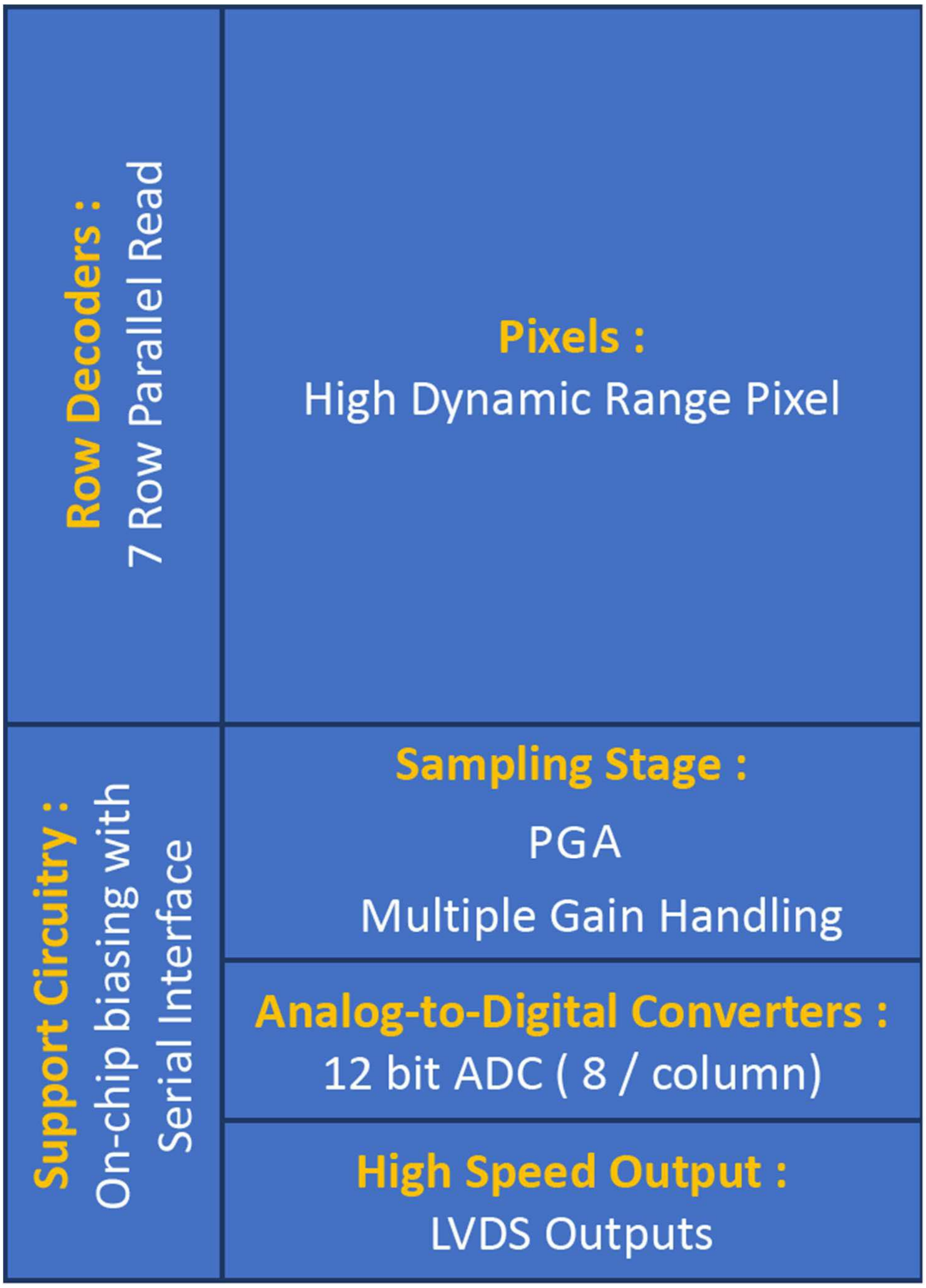

Figure S2 Top-level overview of the ASIC (not to scale)

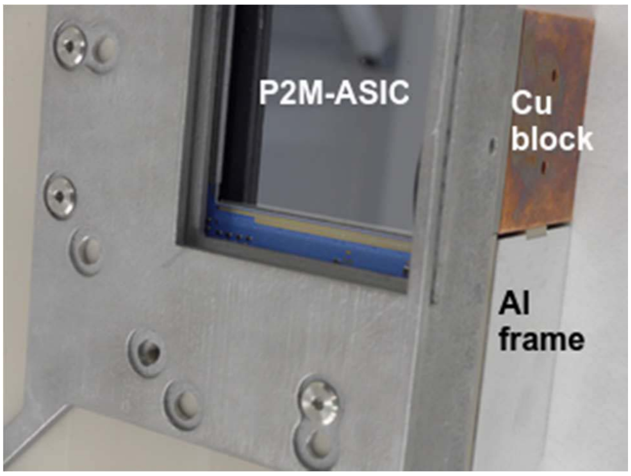

Figure S3 Picture of the P2M cold head. The ASIC can be recognized by its mirror sheen, and part of the wirebond pads on the LTCC are visible. The Molybdenum support under the chip (chosen for CTE match to Si) is hidden by the protective frame, but the Copper block below the Molybdenum support is shown. The Aluminum frame can also be recognized at the side of the Copper block. There is only a small area of contact (using thermally resistive PEEK) between the warm Aluminum frame and the cold Copper block.

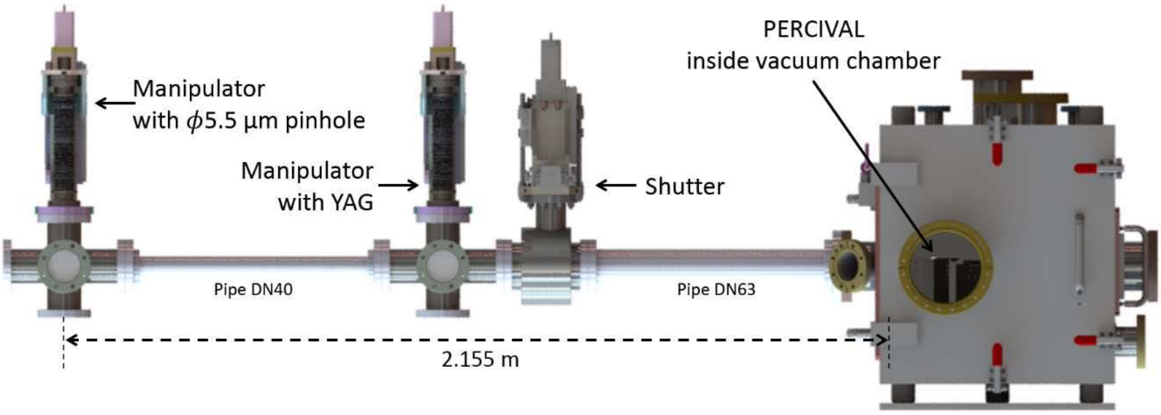

Figure S4 Sketch of the setup for the beamtime at the Variable Polarization XUV Beamline P04 at Petra III.
